# African Jointfir (*Gnetum africanum*) and Editan (*Lasianthera africana*) leaf alkaloid extracts exert antioxidant and anticholinesterase activities in fruit fly (*Drosophila melanogaster*)

**DOI:** 10.1002/fsn3.3307

**Published:** 2023-03-13

**Authors:** Ganiyu Oboh, Ayomide Victor Atoki, Adedayo O. Ademiluyi, Opeyemi B. Ogunsuyi

**Affiliations:** ^1^ Department of Biochemistry Federal University of Technology Akure Nigeria; ^2^ Department of Biochemistry Kampala International University Ishaka Uganda; ^3^ Department of Biomedical Technology Federal University of Technology Akure Nigeria

**Keywords:** alternative experimental model, functional foods, neurodegeneration, nutraceuticals, vegetables

## Abstract

African Jointfir (*Gnetum africanum*) and Editan (*Lasianthera africana*) leaves are two leafy green veggies with several nutritional and medicinal properties. Alzheimer's disease (AD) is a form of neurodegeneration that is believed to cause dementia in affected individuals. The quest for alternative treatments has necessitated the exploitation of plants' secondary metabolites. Plant alkaloids have recently demonstrated relevance in the management of a variety of neurodegenerative disorders; although there is limited information on the neuroprotective properties of alkaloids from various tropical green leafy vegetables with neuroprotective potentials. As a result, this study examined the cholinesterase inhibitory activity and antioxidant potential of alkaloid extracts from the leaves of African Jointfir (*G. africanum*) and Editan (*L. africana*). Standard solvent extraction techniques were used to prepare alkaloid extracts. After that, these extracts were characterized using high‐performance liquid chromatography. In vitro acetylcholinesterase inhibition assay for the extracts was also carried out. Subsequently, the alkaloid extracts were included in the diets of these flies (2 and 10 μg/g) for 7 days. Thereafter, treated fly homogenates were assayed for cholinesterase, monoamine oxidase, and antioxidant enzymes (specifically, glutathione‐S‐transferase catalase, and superoxide dismutase) activities, in addition, thiobarbituric acid reactive substance, reactive oxygen species, and total thiol contents. The extracts showed considerable anticholinesterase, antioxidant, and antimonoamine oxidase capabilities, according to the study's findings. Also, HPLC characterization revealed that desulphosinigrin (597,000 ng/100 g) and atropine (44,200 ng/100 g) are the predominating phytochemicals in Editan and African Jointfir respectively. These extracts could serve as potential sources of nutraceuticals with neuroprotective properties which can be used in the treatment/management of Alzheimer's disease.

## Graphical abstract

This study reveals that alkaloid extracts from Editan (*Lasianthera africana*) and African Jointfir (*Gnetum africanum*) could possess antioxidant and anticholinesterase activities in *Drosophila melanogaster*. Desulphosinigrin and atropine were revealed through high‐performance liquid chromatography to be the predominating plant chemicals in *L. africana* and *G. africanum*, respectively. Alkaloid extracts from both plants exhibited outstanding lipid peroxidative ability.

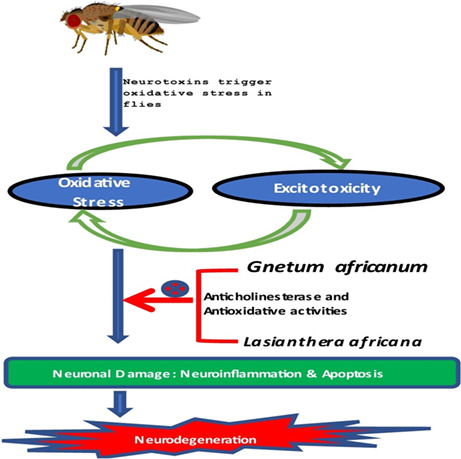

## 1 INTRODUCTION

Neurodegeneration describes the stealthy deterioration in an individual's cognitive function such as memory (Kandlur et al., 2020). This loss is caused by structural changes that do not allow neurons associated with the brain to function normally; or cell death. Neurodegeneration in the form of Alzheimer's disease (AD) is by far the most rampant. This terminal, irremediable, and fatal disease was first identified by Alois Alzheimer in 1906; a German with expertise in psychiatry and neuropathology. The disease was named after him (Pradhan et al., 2022). In spite of AD etiology not being understood in full, acetylcholinesterase (AChE) and butyrylcholinesterase (BChE) inhibition have been adopted as an efficacious preventive and managerial approach of AD treatment (Adeowo et al., 2020). Donepezil, galantamine, tacrine, and rivastigmine are synthetic drugs which are AChE inhibitors used in the treatment of AD; nevertheless, these drugs have limited use because they pose serious side effects, moreover, these drugs are not effective against advanced form of AD. Additionally, these aforementioned drugs do not possess outstanding BChE inhibitory property (Sharma, 2019). As a result, recent studies have channeled energy toward exploring plant compounds as sources of AChE and BChE inhibitors with little to no negative effects that can act as dietary intercessions in the prevention and management of AD (Conforti et al., 2007; Dallanoce, 2022).

Alkaloids are phytochemicals with psychoactive and neuromodulatory properties; extracts of plants rich in alkaloids have been employed in folkloric medicine for the treatment/management of neurodegeneration for centuries with dearth of scientific justifications. Editan (*Lasianthera africana*) and African Jointfir (*Gnetum africana*) leaves are two vegetables which have been reported to be abundant in alkaloids. Therefore, the assessment of alkaloid extracts from these vegetables for their neuroprotective potentials especially in the management of tau‐related neurodegenerative diseases will be highly desirable.

*Lasianthera africana* (LA; Editan) is a perennial, hairless shrub which belongs to the family Icacinaceae. Ethnobotanically, four varieties are known; which possesses distinctive taste, ecological distribution, and leaf color (Bassey et al., 2006). *Lasianthera africana* is widely acceptable as food and more as medicine. It, therefore, plays a crucial role in household food security. From time immemorial, plants have been exploited for the treatment of wide range of diseases by traditional herbalists. According to Ajayi et al. (1989), LA leaves are highly nutritious and can be consumed to alleviate gastrointestinal conditions like diarrhea, constipation, and stomachaches. This claim was supported by Ebana et al. (2016) who reported that LA is rich in phytochemicals that are of nutritional and therapeutic importance. Ekpo et al. (2022) reported that LA extract protected against hepatotoxicity. Unah et al. (2022) reported the fecundity properties of LA in broiler chicken. Other research works reveal that LA possesses antiplasmodial property (Okokon et al., 2007), antimicrobial property (Andy & Ebana, 2019), and antidiabetic property (Nwakaego, 2022). Etukudo (2003) showed that aqueous extract of LA prevented against ingestion, internal heat, and stomach discomfort, when administered orally or enema. The LA leaf's total phenolic and flavonoid content has been documented (Shodehinde et al., 2017). The immunomodulatory and antileishmanial activities of this leaf have also been reported (Okokon et al., 2012). Mineral assessment of LA leaves revealed that it contains a considerable amount of magnesium, potassium, calcium, phosphorus, iron, and vitamins A, B_1_, and C; the leaves were also reported to contains glycoside, saponin, carotenoids, oxalate, and polyphenols in the same study (Wekhe et al., 2022). Recently, Anorue and Ekpo (2020) reported the nonoxidative effect of LA extracts on human hemoglobin.

African Jointfir (*Gnetum africanum*) belongs to the class of vegetables. It is widely consumed in so many African nations, most especially in Nigeria, specifically in the south‐eastern part of the nation, where it is usually called “Okazi.” It is highly cherished for its medicinal property and nutritional value (Cole et al., 2022; Dada et al., 2021; Okerulu & Onyema, 2015). According to reports, this vegetable is high in alkaloids (Ilodibia et al., 2015; Verma, 2020) and it has long been used to treat a variety of illnesses like diabetes, fever, and ulcer (Aborisade et al., 2017). It is used in preparing soups and sometimes consumed as spice (Lalmuanpuii, 2021; Okeke, 2008). Previous studies validated the anti‐inflammatory, hypolipidemic, antioxidative, and hyperglycemic properties of this vegetable (Ilodibia et al., 2015; Ogboye et al., 2018; Okezie et al., 2017). The leaves of African Jointfir is rich in iron, iodine, calcium, and a good source of protein (Dada et al., 2021).

*Drosophila melanogaster*, commonly referred to as fruit fly, has been widely used in biological researches especially in molecular biology and genetics, following its introduction over a century ago (Rocha, 2013). *D. melanogaster* is frequently employed as a model organism in the fields of biochemistry, cell biology, genetics, and molecular biology. Specifically, more than 65%–70% of human disease‐causing genes have been found in these flies (Pandey & Nichols, 2011; Poddighe et al., 2013; Reiter et al., 2001) and hence, it has become a useful tool for studying human disease conditions. Drosophila offers comparative benefits over other models for biological study in that it has a quick generation period, a brief life cycle, and is simple to handle and keep in the laboratory in large numbers (Rocha, 2013).

## 2 MATERIALS AND METHODS

### 2.1 Sample collection

Fresh Editan (*L. africana*) and African Jointfir (*G. africanum*) leaves were obtained from a neighborhood market in Akure, Nigeria, and the Department of Biology at the Federal University of Technology, Akure, Nigeria, performed the authentication. Thereafter, they were dried in air to constant weight and milled. Preceding the extraction of the alkaloids, the milled samples were maintained in a vessel deprived of air.

### 2.2 Drosophila melanogaster stock culture

Drosophila research laboratory, Functional Food and Nutraceutical Unit, Department of Biochemistry, Federal University of Technology Akure, Nigeria provided the wild‐type *D. melanogaster* (Oregon strain) stock culture. The flies were kept and raised on a standard cornmeal medium that has brewer's yeast (1% w/v) and nipagin (0.08% v/w) at constant temperature (25 ± 1°C) and relative humidity (60%) under a 12‐h cycle of darkness and light. The same strain of *D. melanogaster* was used in all the experiments.

### 2.3 Reagents

Chemicals from Sigma Aldrich Co. were purchased, including acetylthiocholine iodide, sulfanilamide, reduced glutathione, and semicarbazide. Sigma Al‐drich, Chemie GmbH provided the trichloroacetic acid (TCA), while BDH Chemicals Ltd. provided the potassium acetate, methanol, acetic acid, hydrochloric acid, aluminum chloride, sodium dodecyl sulfate, hydrogen peroxide, potassium ferricyanide, and ferric chloride. Starch and ascorbic acid were Merck products. All additional chemicals and reagents, with the exception of those noted otherwise, were of analytical quality, and the water was distilled using glass.

### 2.4 Alkaloid extracts preparation

With a few minor adjustments (Ademiluyi, Ogunsuyi, et al., 2016; Ademiluyi, Oyeleye, & Oboh, 2016), the method of Harborne (1981) was used to extract crude alkaloids. In an electrical blender, 350 mL of distilled water and 100 g of pulverized materials were combined in a 1:4 ratio for 5 min. The solutions were filtered using muslin cloth and a Buchner funnel, using a filter paper (Whatman No.1) while operating at a decreased pressure. The supernatants were then transferred to a separating funnel and extracted three times with chloroform after being evaporated at 45°C in a rotary evaporator with drops of 2% sulfuric acid (to make pH = 1). The solutions were divided into two layers: the upper layer was the aqueous layer, and the lower layer was the chloroform layer, which was disregarded. In order to achieve a pH of 9, concentrated ammonium hydroxide was added to this layer. The solutions were then extracted twice with a 3:1 chloroform: methanol mixture and once with chloroform in a separating funnel. The solutions were divided into two layers, with the lower layer being either a layer of pure chloroform or a layer of pure chloroform and methanol, and subsequently evaporated using a rotary evaporator. The residues were extracted using methanol, and finally, the extracts obtained were stored at 13°C in a refrigerator for analysis.

### 2.5 In vitro acetylcholinesterase inhibition assay

The flies were put to sleep in ice before being homogenized with a Teflon homogenizer in 0.1 M phosphate buffer, pH 7.4. The resultant homogenates were spun in a Kenxin refrigerated centrifuge Model KX3400C for 10 min at 10,000 *g*, 4°C (KENXIN Intl. Co.). The supernatant was then removed from the pellet and placed in Eppendorf tubes to be utilized for the tests. This assay was done using a colorimetric technique Ellman et al. (1961). The amount of AChE activity was measured in a reaction mixture that contained 200 mL of an AChE solution (0.415 U/mL in 0.1 M phosphate buffer, pH 8.0), 60 mL of a 5,5‐dithio‐bis (2‐nitro‐benzoic) acid (DTNB) solution (3.3 mM in 0.1 M phosphate‐buffered solution, pH 7.0, containing NaHCO_3_ 6 mM), extract (0–75 μL). Acetylthiocholine iodide (60 L of 8 mM water solution) was added as the substrate after incubation for 20 min at 25°C, and AChE activity was assessed by changes in absorbance (412 nm) within the space of 5 min using UV spectrophotometry at 25°C.

### 2.6 Bioassay

#### 2.6.1 Survival study

The rate of survival of flies exposed to alkaloid extracts from *L. africana* and *G. africana* leaves for 7 days was the subject of the study. Flies (3–5 days old, both sexes) were separated into five groups (*n* = 5), each comprising 40 flies. Group I (Control) flies were placed on basal diet (without alkaloid extracts from leaves), while the Groups II–V were fed with diets complemented with alkaloid extracts from the leaves of *L. africana* and *G. africana* (10–2000 μg/g). The survival rate was calculated by counting the number of dead flies for the first 7 days after the flies were monitored daily for incidence of mortality. Following the treatment time, the data were examined and shown as cumulative mortality and percentage of live flies (Abolaji et al., 2014; Adedara et al., 2016).

#### 2.6.2 Experimental design

Fruit flies (of both sexes, aged 3–5 days) were grouped into 5 with 40 flies per vial (*n* = 5). Group I was fed normal diet, groups II and III were fed normal diet containing alkaloid extracts of *L. africana* (2.0 and 10 μg/g), and groups IV and V were given normal diet containing alkaloid extracts of *G. africana* (2.0 and 10 μg/g). The selection of alkaloid extract concentrations was based on a survival analysis that demonstrated that the chosen concentrations did not significantly increase fly mortality (Figures S1 and S2).

#### 2.6.3 Sample preparation for biochemical assays

The weight of the flies was determined after being put to sleep in ice. The head section was cautiously removed, after which it was homogenized in 10 volumes of buffer (specifically, 0.1 M phosphate), pH 7.4, and centrifuged in a Kenxin refrigerator centrifuge at 10,000 *g* for 10 min (Model KX3400C; KENXIN Intl. Co.). The supernatants were gathered into labeled Eppendorf tubes and utilized to measure the biochemical parameters afterwards. Total protein content of fly homogenates was measured by the Coomassie blue technique according to Bradford (1976) while bovine serum albumin (BSA) serves as standard. In three separate studies, copies of every biochemical determination were performed.

#### 2.6.4 Lipid peroxidation and thiobabituric acid reactions

This was carried out using the technique described by Ohkawa et al. (1979) with slight modifications as recently reported by Ogunsuyi, Ademiluyi, and Oboh (2020) and Ogunsuyi, Oboh, et al. (2020). In a nutshell, 0.05 mL of tissue homogenate was reacted with 0.15 mL of sodium dodecyl sulfate (SDS) at 8.1%, 0.25 mL of HCL/acetic acid (pH = 3.4), and 0.25 mL of thiobarbituric acid (TBA), and the combination was then incubated at 100°C for an hour. Using a spectrophotometer, the resultant species of reactive thiobarbituric acid were measured at 532 nm and expressed as malondialdehyde equivalents.

#### 2.6.5 Total thiol content determination

Determination of the amount of total thiol in the fly homogenate was carried out by the technique described by Ellman (1959) as reported by Abolaji et al. (2017). Twenty microliters of homogenate, 10 μL of 10 mM DTNB, and 270 μL of 0.1 M potassium phosphate buffer (pH 7.4) made up the reaction mixture. The absorbance was measured at 412 nm after a 30‐min incubation period at room temperature. Following the calculation of total thiol content, the amount of total thiol in the fly tissues was presented as μmol GSH/mg protein.

#### 2.6.6 Glutathione‐S‐transferase activity estimation

The Habig and Jakoby (1981) method was used to measure glutathione‐S‐transferase (GST) activity with slight modifications using 1‐chloro‐2,4‐dinitrobenzene (CDNB) as a substrate. 20 μL of sample (1:5 dilution), 10 μL of 25 mM CDNB, and 270 μL of a solution containing (20 mL of 0.25 M potassium phosphate buffer, pH 7.0, 10.5 mL of water that was distilled, and 500 mL of 0.1 M GSH at 25°C) made up the reaction mixture. Using the molar extinction coefficient (*ε*) of 9.6 mM/cm for CDNB conjugate, the reaction was monitored for 5 min (30‐s intervals) at 340 nm in a SpectraMax plate reader (Molecular Devices). GST activity was then expressed as mol/min/mg protein.

#### 2.6.7 Catalase activity determination

According to a recent publication by Ogunsuyi, Ademiluyi, and Oboh (2020) and Ogunsuyi, Oboh, et al. (2020) the activity of catalase (CAT) in the fly tissue homogenate was measured using the technique described by Sinha (1972). Briefly stated, 1.0 mL of 0.01 M phosphate buffer (pH = 7.0) was added to 0.1 mL of each tissue homogenate sample before reacting with 0.4 mL of 2 M H_2_O_2_. The addition of 2.0 mL of the dichromate/acetic acid solution was used to halt the process. Using a spectrophotometer, the reaction mixture's absorbance was measured at 620 nm. In the presence of 1.0 mL of 0.01 M sodium phosphate buffer, 0.4 mol of 2 M H_2_O_2_ was combined with 2 mL of dichromate/acetic acid solution to create a standard curve (pH 7.0). Following that, the activity of catalase was determined and presented as U/g/mg protein.

#### 2.6.8 Superoxide dismutase activity determination

The Alía et al. (2003) method was used to determine superoxide dismutase (SOD). A mixture of 1.0 mL of 50 mM carbonate buffer (pH 10.2) and 0.017 mL of adrenaline (0.06 mg/mL) was added to an aliquot of 0.05 mL tissue homogenate. Using a spectrophotometer, the absorbance was taken at 480 nm for 2 min at intervals of 15 s. The units of SOD activity per mg of protein were used to express SOD activity.

#### 2.6.9 Cholinesterase activity assay

Using substrates for both acetylcholinesterase and butyrylcholinesterase, the activity of cholinesterase (ChE) was assayed using the colorimetric Ellman's technique, reported by Oboh et al. (2012) 1.4 mM acetylthiocholine iodide or butyrylthiocholine iodide, 15 μL of homogenate, 100 μL of distilled water, 100 μL of 100 mM sodium phosphate buffer (pH 7.4), and 1.7 mM of DTNB made up the reaction mixture. Acetylcholinesterase and butyrylcholinesterase activities were expressed as mmolAcSch/h/mg protein or mmolBuSch/h/mg protein, respectively, measured at 412 nm.

#### 2.6.10 Assay for monoamine oxidase activity

The monoamine oxidase (MAO) activity was determined as previously reported (McEwen, 1965). 200 μL of fly tissue, 0.5 μmol/mL of benzylamine, 400 μL of 100 mM phosphate buffer (pH 7.4), and 1.3 mL of distilled water constituted the reaction mixture. The reaction mixture was incubated for 30 min at 25°C after which 1 mL of perchloric acid (10%) was added. Following this step, the reaction mixture was spun at 1500 g for 10 min. The activity of MAO was then measured at 280 nm and presented as mmol/mg protein.

#### 2.6.11 Estimation of reactive oxygen species concentration

The Hayashi et al. (2007) method was used to measure the level of reactive oxygen species (ROS).

In a nutshell, 1400 μL of sodium acetate buffer and 50 μL of tissue homogenate were made available in a cuvette. Then, one thousand microliter of n‐n‐diethyl‐para‐phenylenediamine (DEPPD) reagent mixture (6 mg/mL of DEPPD with 4.37 M of ferrous sulphate dissolve in 0.1 M sodium acetate pH 4.8) was added, and it was incubated at 37°C for 5 min.

A spectrophotometer was used to test the absorbance at 505 nanometers. From an H_2_O_2_ calibration curve, ROS levels in the tissue were estimated and expressed as Unit/mg protein.

#### 2.6.12 HPLC characterization of alkaloid

In a borosilicate beaker with 10 mL of 70% methanol, 1 g of the powdered samples was added. The sample combination was extracted in a room temperature ultrasonic bath for 20 min. Following extraction, the sample combination was extracted and then spun twice for 10 min at 11,200 *g* A 0.22 membrane filter was used to collect and filter the supernatant. For calibration and establishing correlation coefficients, the standards of different concentrations were made ready for introduction into the HPLC system. The same process used to inject standard mixtures into the HPLC machine was used to inject samples.

### 2.7 Data analysis

The data were presented as mean ± standard deviation (SD) and suitably evaluated using one‐way analysis of variance (ANOVA) and a subsequent Tukey's post hoc test. GraphPad PRISM software (V.5.0) was used for all statistical analyses.

## 3 RESULTS

**FIGURE 1:**
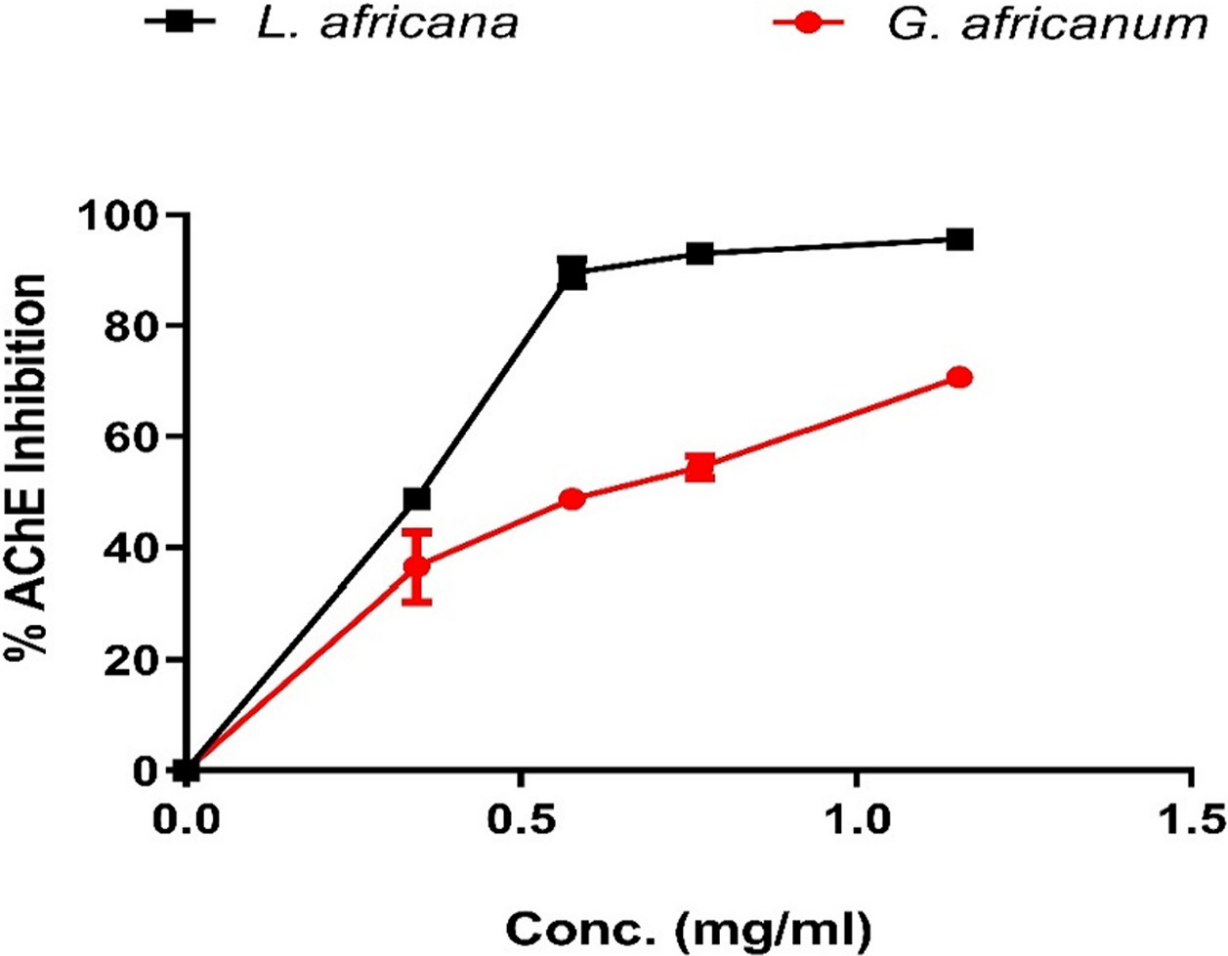
In vitro acetylcholinesterase (AChE) inhibitory effect of alkaloid extracts of *Lasianthera africana* and *Gnetum africanum* leaves in *Drosophila melanogaster*.

The result presented in Figure 1 revealed that both alkaloid extracts inhibited acetylcholinesterase (AChE) in a dose‐dependent manner in the range 0–1.154 mg/mL.

**FIGURE 2:**
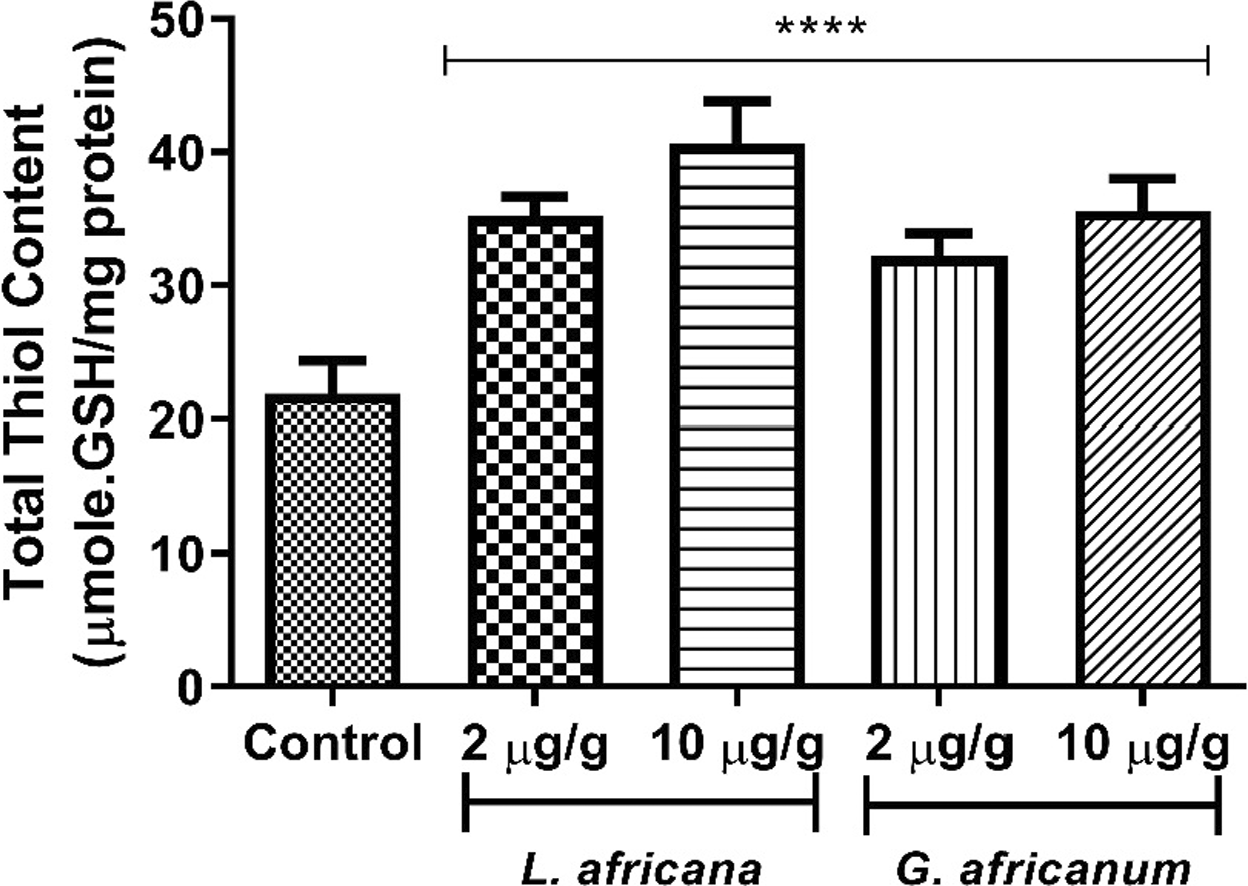
Effect of alkaloid extracts of *Lasianthera africana* and *Gnetum africanum* leaves on Total Thiol level in *Drosophila melanogaster*. Bars represent mean ± standard deviation (*n* = 5). Mean values are significantly different at ****p < .0001 compared to control.

The effect of LA and GA alkaloid supplemented diet on total thiol content in *D. melanogaster* is presented in Figure 2. The alkaloid extracts increased total thiol content across all concentrations tested when compared with the control.

**FIGURE 3:**
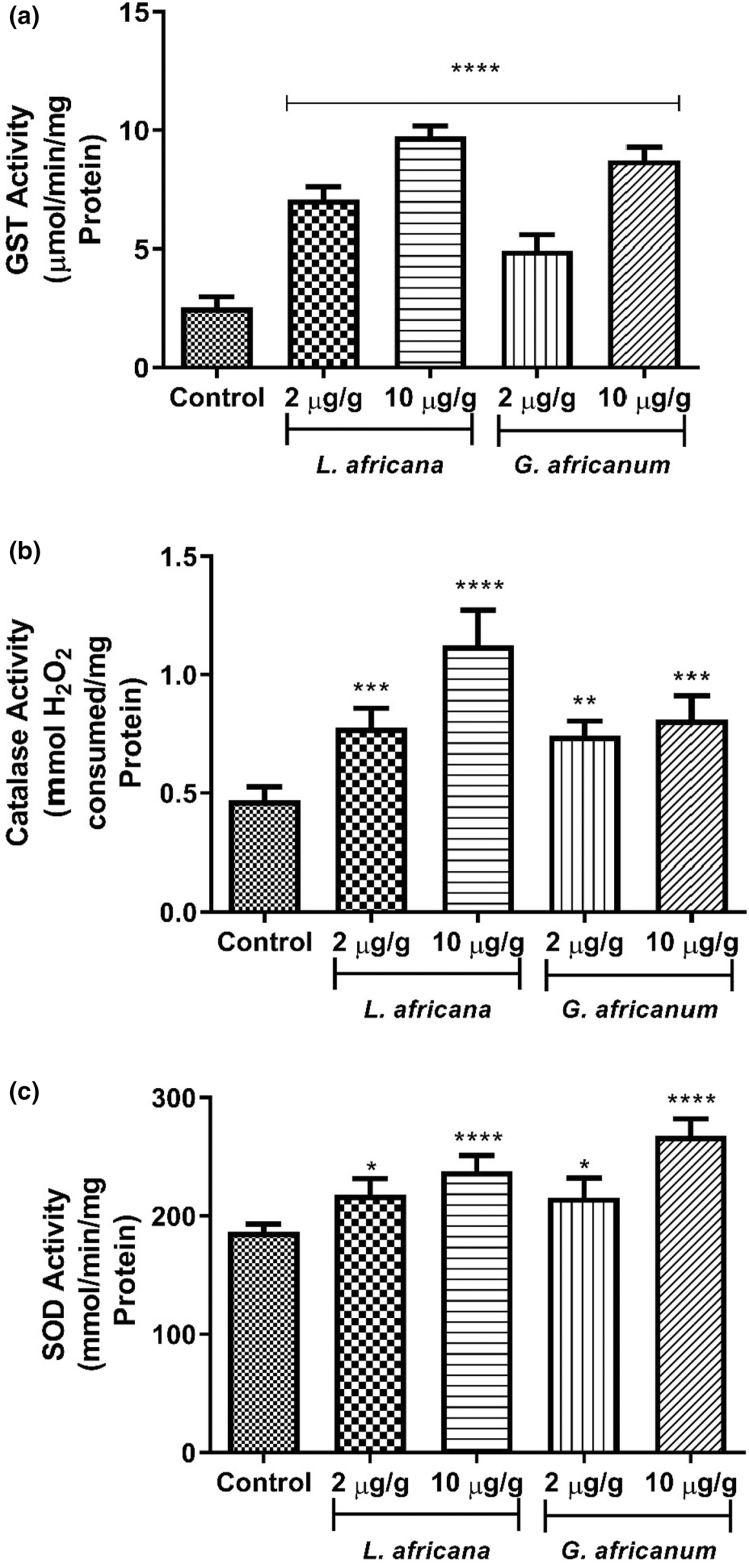
(a) Effect of alkaloid extracts of *Lasianthera africana* and *Gnetum africanum* leaves on the activity of Glutathione‐S‐transferase in *Drosophila melanogaster*. Bars represent mean ± standard deviation (*n* = 5) Mean values are significantly different at ****p < .0001 compared to control. (b) Effect of alkaloid extracts of *L. africana* and *G. africanum* leaves on catalase activity in *D. melanogaster*. Bars represent mean ± standard deviation (*n* = 5). Mean values are significantly different at **p < .01; ***p < .001; ****p < .0001 compared to control. (c) Effect of alkaloid extracts of *L. africana* and *G. africanum* leaves on superoxide dismutase (SOD) in *D. melanogaster*. Bars represent mean ± standard deviation (*n* = 5). Mean values are significantly different at *p < .05; ****p < .0001 compared to control.

The effect of LA and GA alkaloid supplemented diet on GST activity in *D. melanogaster* is presented in Figure 3a. Both extracts caused a considerable increase in the GST activity of flies when compared with the control group.

The effect of LA and GA alkaloid supplemented diet on catalase activity in *D. melanogaster* is presented in Figure 3b. Both extracts caused a considerable increase in the catalase activity in flies when compared with the control group.

The effect of LA and GA alkaloid supplemented diet on superoxide dismutase activity in *D. melanogaster* is presented in Figure 3c. Both extracts caused a considerable increase in the superoxide dismutase activity of flies when compared with the control group.

**FIGURE 4:**
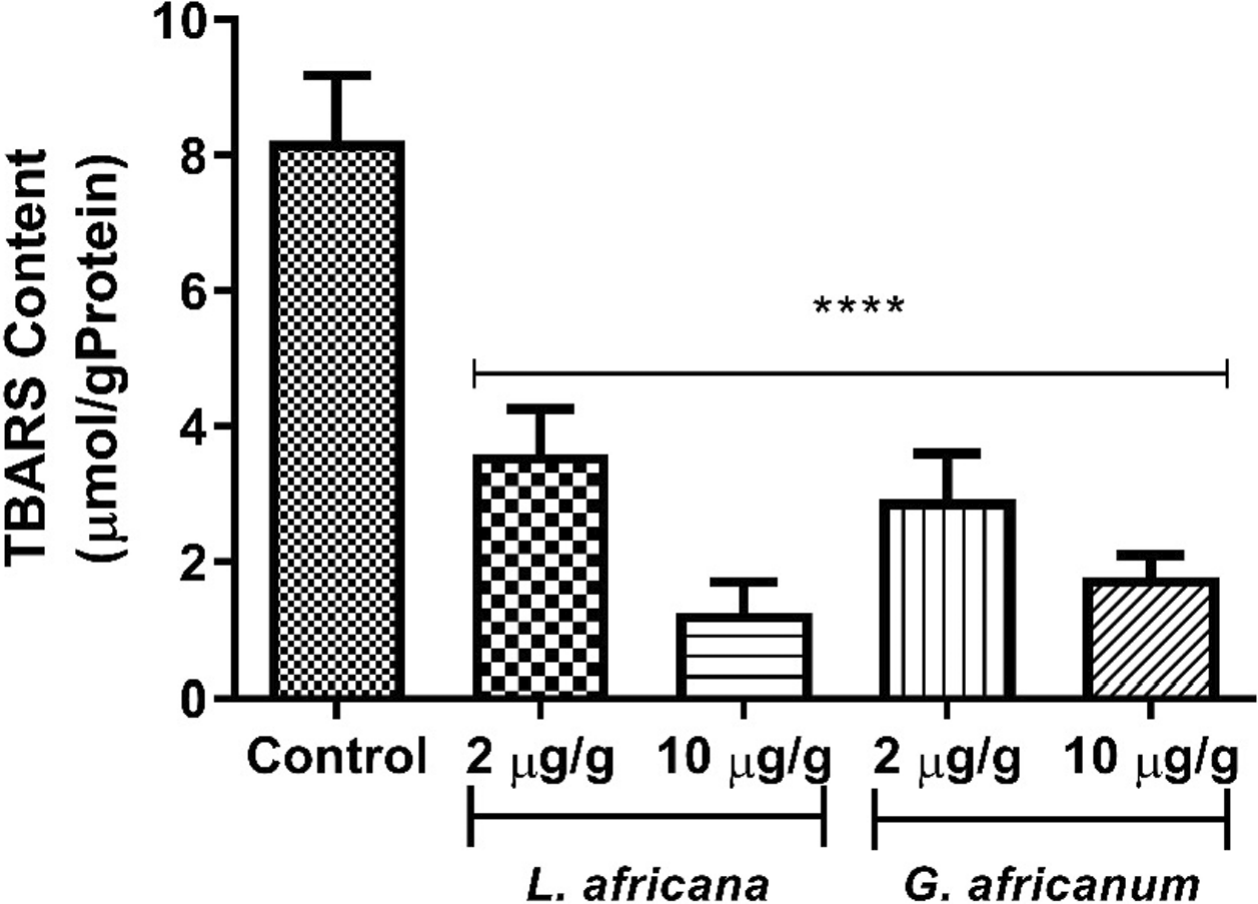
Effect of alkaloid extracts of *Lasianthera africana* and *Gnetum africanum* leaves on thiobarbituric acid reactive species (TBARS) level in *Drosophila melanogaster*. Bars represent mean ± standard deviation (*n* = 5). Mean values are significantly different at ****p < .0001 compared to control.

The ability of LA and GA alkaloid extracts to curtail lipid peroxidation in flies, in vivo is presented in Figure 4. Both alkaloid extracts inhibited TBARS production in fly homogenate. However, LA alkaloid extract appears to have a higher TBARS inhibitory activity than GA alkaloid extract.

**FIGURE 5:**
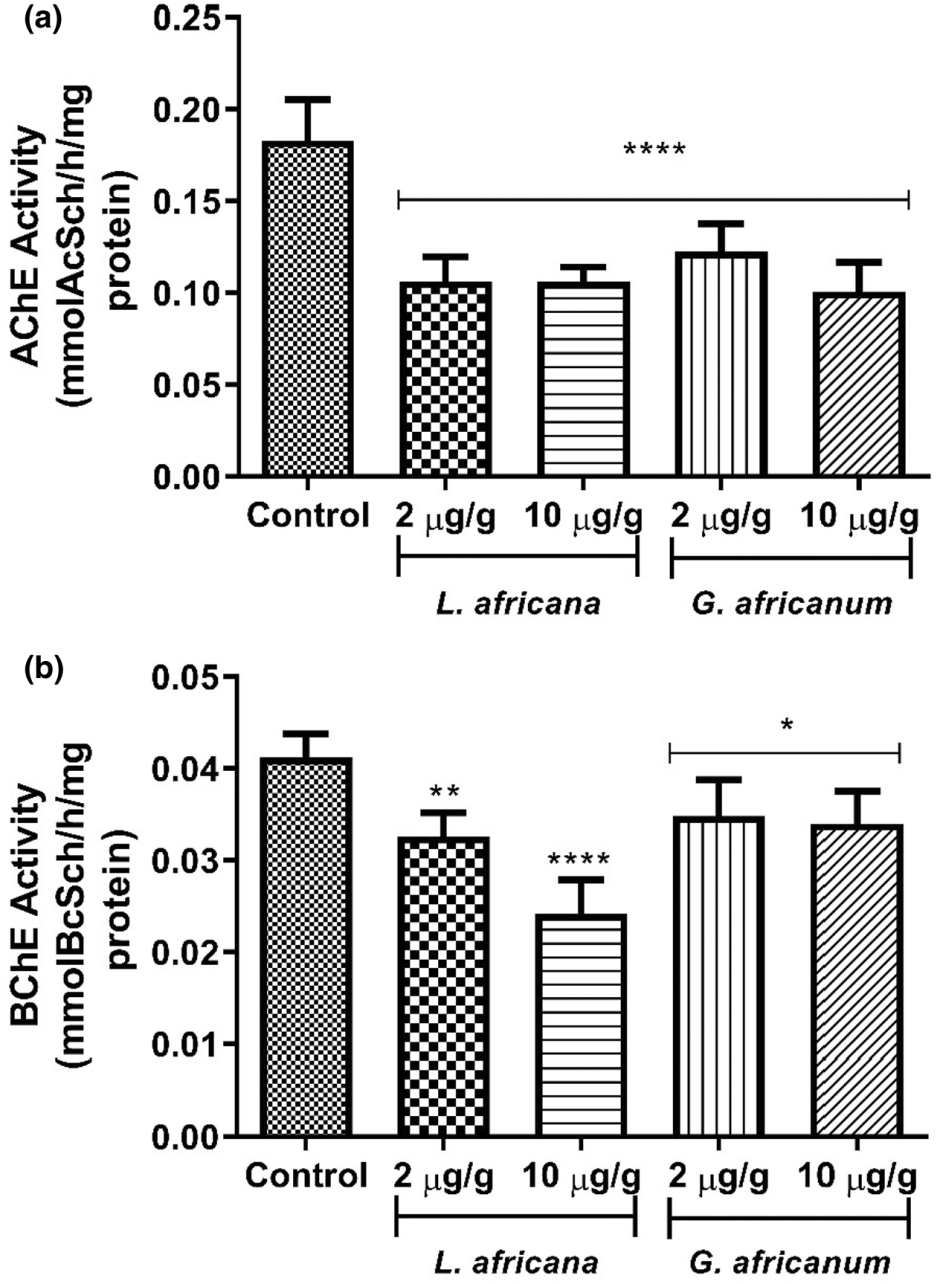
(a) Effect of alkaloid extracts of *Lasianthera africana* and *Gnetum africanum* leaves on cholinesterase activity (using acetylthiocholine iodide as substrate) in *Drosophila melanogaster*. Bars represent mean ± standard deviation (*n* = 5). Mean values are significantly different at ****p < .0001 compared to control. (b) Effect of alkaloid extracts of *L. africana* and *G. africanum* leaves on cholinesterase activity (using butyrylthiocholine iodide as substrate) in *D. melanogaster*. Bars represent mean ± standard deviation (*n* = 5). Mean values are significantly different at *p < .05; **p < .01; ***p < .0001 compared to control.

The inhibitory effect of LA and GA alkaloid extracts on AChE activity is presented in Figure 5a. Both alkaloid extracts inhibited AChE activity considerably, when compared to the control group.

The inhibitory effect of LA and GA alkaloid extracts on BChE activity is presented in Figure 5b. Both alkaloid extracts inhibited BChE activity considerably, when compared to their respective control groups.

**FIGURE 6:**
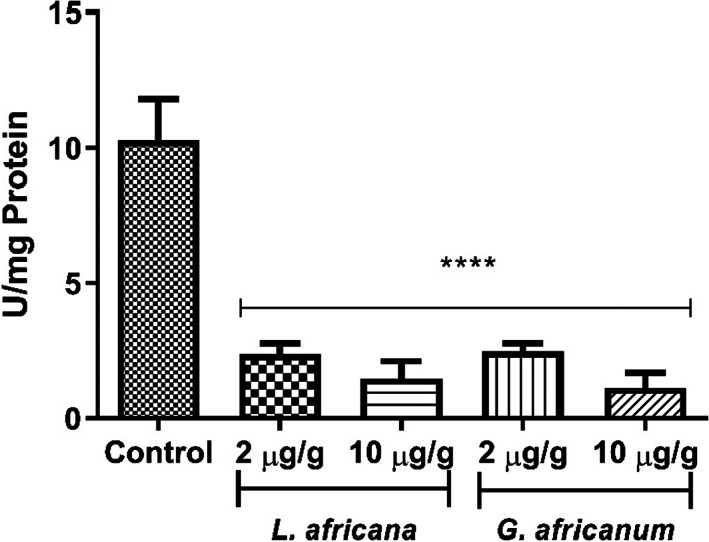
Inhibitory effect of alkaloid extracts of *Lasianthera africana* and *Gnetum africanum* leaves on Monoamine oxidase activity in *Drosophila melanogaster*. Bars represent mean ± standard deviation (*n* = 5). Mean values are significantly different at ****p < .0001 compared to control.

The inhibitory effect of LA and GA alkaloid extracts on MAO activity was also investigated in this study, as shown in Figure 6. Both alkaloid extracts inhibited MAO activity in the flies.

**FIGURE 7:**
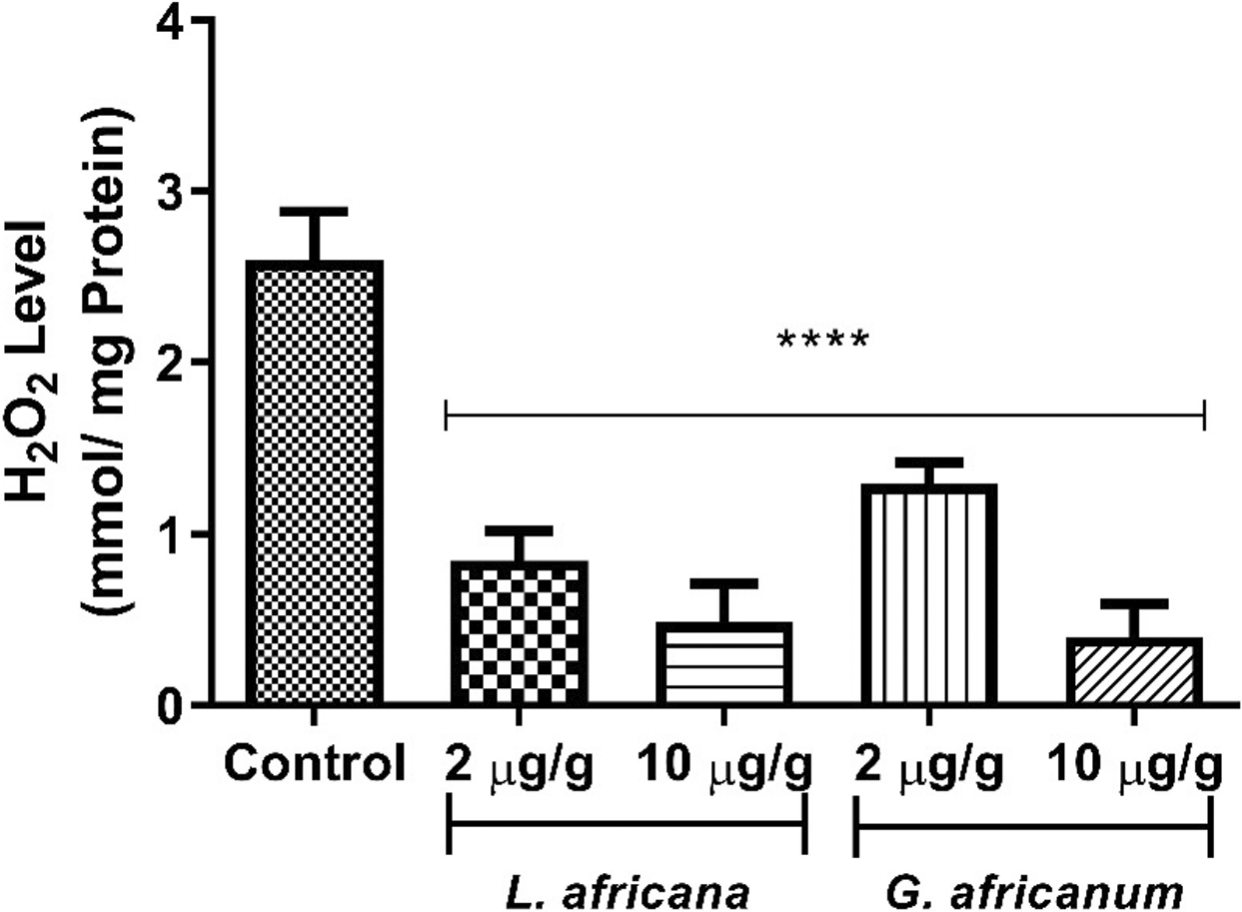
Effect of alkaloid extracts of *Lasianthera africana* and *Gnetum africanum* leaves on reactive oxygen species level (ROS) in *Drosophila melanogaster*. Bars represent mean ± standard deviation (*n* = 5). Mean values are significantly different at ****p < .0001 compared to control.

The effect of LA and GA alkaloid supplemented diet on reactive oxygen species (ROS) in *D. melanogaster* is presented in Figure 7. Both extracts caused a significant decrease in the level of ROS in the flies when compared with the control group.

## 4 DISCUSSION

Functional foods and nutraceuticals are gaining scientific popularities for their numerous health benefits including neuroprotective properties (Daliri & Lee, 2015). The geometric increase in morbidity and mortality associated with AD and other forms of age‐related dementia across the globe has necessitated the interest about the role of phytochemical in the amelioration of neurodegenerative disorders. Accordingly, the understanding of the biochemical rationale behind the neuroprotection conferred by phytochemicals is crucial to developing new approaches and strategies of addressing neurodegeneration. The use of invertebrates in understanding pathophysiological conditions and alterations have gained popularity in the last 10 years. Fruit fly has been used as a complementary and sometimes as an alternative model organism to vertebrates in studying human pathologies. In this research, to explore the potential neuroprotective capabilities of alkaloid extracts from *L. africana* and *G. africanum*, *D. melanogaster* flies were used as the experimental organism.

Cholinergic neurotransmission is largely facilitated by acetylcholine, through acetylcholine receptors' upregulation. AChE inhibitors improve the cholinergic neurotransmission by slowing down the degradation of ACh. In this present study, it was observed (in vitro) that the alkaloid extracts from both alkaloid extracts significantly inhibited AChE activity (Figure 1) dose dependently (0–1.15 mg/mL), with LA showing significantly higher inhibition (EC_50_ = 0.39 mg/mL) compared to GA extract (EC_50_ = 0.70 mg/mL). To correlate this result in vivo, it was also observed (Figure 5a,b) that LA and GA alkaloid extracts supplemented diets (2.0 and 10 μg/g), brought about a decrease in cholinesterase activities using both acetylthiocholine iodide and butyrylthiocholine iodide as substrates. This correlate with the reports of Oboh et al. (2019) where alkaloid extracts were found to exert anticholinesterase activity in vitro. Acetylcholinesterase (AChE) inhibitors still remain the “gold standard” for the management of mild‐to‐moderate AD. Furthermore, it has been shown that pronounced inhibition of butyrylcholinesterase (BChE) activity is looked for in humans, simply because hydrolytic activity of BChE predominates as cholinesterase in late‐stage AD in humans (Ademosun & Oboh, 2014). Additionally, according to Ballard et al. (2002), BChE inhibition reduces the accumulation of neurotoxic plaques seen in the advanced stage of AD. This finding is also in agreement with previous observation in *D. melanogaster* where alkaloid extracts was shown to decrease AChE activity (Ogunsuyi, Ademiluyi, & Oboh, 2020; Ogunsuyi, Oboh, et al., 2020). It is also in accordance with previous study of Akinyemi et al. (2017) where curcumin was shown to exhibit a significant anticholinesterase activity in *D. melanogaster*. This is also in agreement with previous observation in which alkaloids/plant alkaloid extracts exhibit AChE and BChE inhibition in mammalian experimental models (Mythri & Srinivas Bharath, 2012; Shen, Li, et al., 2013; Shen, Xiao, et al., 2013). Therefore, the decrease in cholinesterase activity after exposing flies to alkaloid extract could result in an increase in acetylcholine concentration and subsequent increase in acetylcholine bioavailability in the synaptic cleft, ultimately increasing the efficiency of cholinergic neurotransmission in the flies (Akinyemi et al., 2018; Williams, 2017). Therefore, the observed reduction in cholinesterase activity in the flies treated with alkaloid extracts suggests their potential anticholinesterase properties especially as nutraceuticals and diet supplements to treat neurodegenerative diseases, particularly AD.

Monoamine oxidase (MAO) is a flavin‐containing amine oxidoreductase that catalyzes the oxidation of monoamine, employing oxygen to clip off their amine group (Edmondson et al., 2004; Tipton et al., 2004). Oxidation of monoamine neurotransmitters by MAO impairs monoaminergic neurotransmission. Inhibiting MAO activity could, therefore, be a therapeutic strategy for treating or preventing neurodegenerative disorders, particularly AD and PD (Lühr et al., 2010). In the brain of neurodegenerative disease patients, it has been shown that MAO activity is increased; and the level of this increase correlates with the severity of the disease (Stamer et al., 2002). Previous studies reported that alkaloid extracts are potent MAO inhibitors (Ademiluyi, Ogunsuyi, et al., 2016; Ademiluyi, Oyeleye, & Oboh, 2016; Baek et al., 2018). Study carried out by Oboh et al. (2019) also revealed that alkaloid possesses anti‐monoamine oxidase property. This is consistent with the findings of this investigation (Figure 6) in which LA and GA alkaloid extracts‐fed flies observed significantly lower MAO activities compared to control flies suggesting their MAO inhibitory properties. In addition, for each of the samples, we observed a significantly lower MAO activity in flies fed higher concentration of the extracts; however, no discernible difference was found between the MAO activities of both samples (2.0 and 10 μg/g). It is, therefore, wise to assume that substantial amount of monoaminergic neurotransmitter, such as dopamine, associated with specific region of the brain of these flies, will be found at “dangerously” low concentration. Dopamine has been reported to regulate motor function (Salamone et al., 2012; Vallone et al., 2000). Therefore, consequent decrease in the level of monoaminergic neurotransmitters, coupled with attendant significant (*p* < .05) decrease in the activities of cholinesterases suggest these alkaloid extracts as a suitable nutraceutical candidates.

Monoamine oxidase activity has been shown to be accompanied by an increase in reactive oxygen species and thus, their inhibition suggests reduction in the attendant production of ROS (Pizzinat et al., 1999). Therefore, the alkaloid extracts' ability of LA and GA to reduce MAO activity, coupled with their antioxidant properties further suggests their neuroprotective properties. The result of this present study clearly reveals that dietary supplementation of LA and GA alkaloid extracts improve antioxidant status in flies, as evident by the decrease in the level of lipid peroxidation and ROS production as shown in Figures 4 and 7, respectively. This is consistent with earlier research in which dietary plant alkaloids exhibited antioxidant properties in *D. melanogaster* by reducing levels of lipid peroxidation and ROS levels (Agunloye et al., 2021; Oboh, Ogunsuyi, Awonyemi, & Atoki, 2018; Oboh, Ogunsuyi, Ojelade, & Akomolafe, 2018). In this study, ROS was quantified as H_2_0_2_ equivalence. H_2_0_2_ is capable of eliciting the production of highly reactive hydroxyl radical under the right redox environment and in turn, OH elicits deleterious oxidative damages to biomolecules including protein decarboxylation, DNA damage, and lipid peroxidation (Cadet et al., 1999; Du & Gebicki, 2004). Therefore, it is hypothesized that the alkaloid extracts in this study could attenuate H_2_0_2_ production by different mechanisms including increasing H_2_0_2_ breakdown or scavenging OH radicals. It is, therefore, interesting to note in Figure 3b that flies fed dietary inclusions of LA and GA significantly increased catalase activity (Figure 3b). This is in agreement with earlier report of Oboh, Ogunsuyi, Awonyemi, and Atoki (2018) and Oboh, Ogunsuyi, Ojelade, and Akomolafe (2018) where alkaloid extracted from African Jointfir was shown to improve antioxidant status in *D. melanogaster* by increasing catalase activity. Catalase is an antioxidant enzyme that breaks down H_2_0_2_ into water, and works in tandem with superoxide dismutase (SOD), the enzyme that carry our dismutation of superoxide anion into H_2_0_2_ (Chang et al., 2014). Therefore, it is significant to observe in Figure 3c that in correlation with catalase activity, dietary inclusions of LA and GA alkaloid extracts also increase SOD activity further corroborating their antioxidant properties. Shen, Li, et al. (2013) and Shen, Xiao, et al. (2013) reported that *D. melanogaster* has a region that encodes enhancement of SOD activity and four regions that encode suppression of SOD activity. Disruption of gene encoding SOD in *D. melanogaster* has been shown to reduce SOD activity (Paul & Duttaroy, 2003; Woodruff‐Pak et al., 2001). Overexpression of this gene, however, resulted in increased SOD activity in *D. melanogaster* (Aigaki et al., 2002; Sun et al., 2004). Therefore, the alkaloid extracts' ability to improve the activity of these flies could be associated with the alkaloids' potential to enhance the expression of the gene encoding SOD activity.

While catalase and SOD catalyzes the neutralization of intracellular peroxides and ROS, respectively, GST is a phase II detoxification antioxidant enzyme that catalyzes hydrolysis of electrophilic oxidants; by conjugating glutathione with electrophilic centers of these oxidants. GST has different isoforms; it has also been shown to participate in activities that improve cell survival to genotoxic substances and oxidative stress. In this study, it was observed that control flies exhibited a marked reduced GST activity compared to treated flies. This strongly shows that under the experimental conditions, this phase II detoxification enzyme was not active. Given that GST has several isoforms, it is possible that some isoforms increased in activity while more of the isoforms had a decreased activity, thus, leading to an overall reduction in GST activity. This present study reveals that dietary supplementation of LA and GA alkaloid extracts improve antioxidant status in flies across all concentration tested, as evident by increased activity of GST (Figure 3a). This property could be largely attributed to the antioxidative potential of LA and GA alkaloid extracts—the protection offered by these extracts in the analyzed concentration could be associated with the properties of the inherent alkaloid. At all concentrations tested, LA alkaloid extract proved more ameliorative than its GA counterpart. Therefore, it could be said that inherent compounds such as desulfosinigrin (being the most abundant compound in LA), piperidine, piperine, ephedrine, vernomine, powelline, lactucin, akuammidine, mitraphylline, and echitamine (as shown in Table 1a) found in LA alkaloid extract are responsible for this pharmacological effect. Mechanism‐wise, the inhibition of Keap1 which led to the activation of Nrf2 signaling pathway could be stated as being the mechanism that confers unto these compounds the ability to improve antioxidant status (Ecker et al., 2017).

**TABLE 1:** HPLC characterization of constituent alkaloids in Editan (*Lasianthera africana*) and African Jointfir (*Gnetum africanum*) leaves extract.

Editan (*Lasianthera africana*)	RT (min)	Amount (ng/100 g)	African Jointfir (*Gnetum africanum*)	RT (min)	Amount (ng/100 g)
Desulphosinigrin	14.154	597,000	Atropine	12.441	44,200
Piperidine	16.008	3720	Scopolamine	14.205	17,200
Piperine	17.179	154	Solasonine	16.108	1.76
Ephedrine	17.834	245	Alpha‐Chaconine	18.774	12,100
Vernomine	18.1	2.15	Solanidine	21.524	792
Powelline	18.673	47.4	Demissine	23.887	78.9
Lactucin	19.586	0.0309			
Akuammidine	26.925	0.414			
Mitraphylline	27.594	3.28			
Echitamine	27.885	1.06			

Glutathione‐S‐transferase catalytic activity depends solely on the availability of glutathione, the most abundant low‐molecular‐weight endogenous thiol (Hellou et al., 2012). Additionally, measuring endogenous thiols, which are a reflection of chemical changes in the thiol groups of peptides and proteins, is a secondary method of measuring oxidative stress. Total thiol content of control flies is observed to be significantly (*p* < .05) low, in comparison with the treated flies (Figure 2). However, LA and GA alkaloid extracts proved ameliorative at all concentration tested, by restoring thiol content in these flies. The increase in thiol levels in the treated flies could hence, justify the increase in GST activities observed in the same group of flies. This outcome is consistent with Oboh, Ogunsuyi, Awonyemi, and Atoki (2018) and Oboh, Ogunsuyi, Ojelade, and Akomolafe (2018) where bitter kola was shown to improve thiol level in *D. melanogaster*.

## 5 CONCLUSION

In summary, this study shows that alkaloid extracts from LA and GA could possess antioxidant and anticholinesterase activities in *D. melanogaster*. Inherent alkaloid extracts of *L. africana* and *G. africanum* were revealed through high‐performance liquid chromatography. Data presented herein support the consumption of LA and GA as functional foods. Further studies on co‐administration of these alkaloids are, however, recommended to investigate their possible synergistic capabilities/effects.

## FUNDING INFORMATION

The World Academy of Sciences (TWAS) Grant No. 16‐500 RG/CHE/AF/AC_G‐FR3240293300 provided funding for this study.

## CONFLICT OF INTEREST STATEMENT

The authors declare no conflict of interest.

## ACKNOWLEDGEMENTS

The authors acknowledge *Drosophila Research Laboratory, Functional Food and Nutraceutical Unit, Federal University of Technology, Akure, Nigeria for providing the facilities used in undertaking this study.*

## Supporting information


Figure S1.
Figure S2.

## Data Availability

Data available on request from the authors.
