# Supplementary material for: African Jointfir (Gnetum africanum) and Editan (Lasianthera africana) leaf alkaloid extracts exert antioxidant and anticholinesterase activities in fruit fly (Drosophila melanogaster)
Source: Food Sci Nutr. 2023 Mar 13;11(6):2708–18. doi: 10.1002/fsn3.3307 (PMC10261729; doi:10.1002/fsn3.3307)
Supplement: Supplementary file 1 — Figure S1. Figure S2. [file FSN3-11-2708-s001.docx]

(i)

(ii)

**Supplementary** **Figure 1**: Effect of alkaloid extract of *Lasianthera africana* leaf on: **(i)** survival and **(ii)** day 7 survival rate in *Drosophila melanogaster*. Bars represent mean ± standard deviation. Mean values are significantly different at *P* < 0.05* compared to control

(i)

(ii)

**Supplementary Figure 2**: Effect of alkaloid extract of *Gnetum africanum* leaf on: **(i)** survival and **(ii)** day 7 survival rate in *Drosophila melanogaster*. Bars represent mean ± standard deviation. Mean values are significantly different at *P* < 0.05* compared to control
